# Hospitals of the World

**Published:** 1893-03-11

**Authors:** 


### HOSPITALS OF THE WORLD.*

### I.—EARLY HOSPITALS IN EUROPE.

Many causes have contributed to the founding and perfecting of hospital systems as they may be studied to-day in the various countries of Europe. But in the main the buildings and their endowment are seen to be due to one of four agencies—private benevolence, a religious order, a community of laymen, or the action of Government. To the wise generosity of one man or woman is owing the existence of some of the most famous hospitals in our own and other lands. In Rome the duty of caring for the sick was early recognised, and after the accession of the Christain Emperor Constantine the scattered efforts of the clergy became systemised, and many charitable institutions were founded and endowed. "Fabiola, a Roman lady of the ancient line of Fabius, sold her very considerable patrimony, and besides helping the poor, founded the first hospital in Rome, in connection with which there was an institution for convalescents in the country. She admitted the sick whom she collected from the public roads. She constituted herself a nurse, the first of her order, and was in the habit of bearing the sick about and of bathing their sores on which others would not look. No less generous of her person than of her purse, she braved discomforts that would have discouraged others, and seemed to feel that in caring for the wounds of her patients she was caring for those of her Saviour." The Hospital of the Holy Spirit, at this day doing good work in Rome, illustrates in the course of its long career the working of the four agencies to which we have alluded. It originated in the eighth century among the community of Anglo-Saxons who had made a settlement in Rome. It had apparently fallen into decay when Innocent III. rebuilt it in 1203, and put it under the direction of the Order of the Holy Spirit, founded by Guido for the care of the sick some years previously. After the death of Guido this hospital became the parent institution of the order, in place of the one originally founded by him at Montpellier. Again rebuilt by Sixtus IV., it forms, with its striking octagonal dome, one of the best examples of the early Renaissance in Rome. And now, in common with other Opere Pie, or charitable institutions in Italy, it has passed under the control of the Government.

More than one hospital in Rome was founded among special trades for the aid of their members, and partook, therefore, of the nature of a benefit society. "The better sort of bakers built a church and hospital to Our Lady of Loretto. 'The hospital is common to all afflicted with sores and fevers, but more particularly designed for bakers.'" In 1580 "the coachmen and carmen joined together, and out of themselves instituted a sodality of the better sort, which built an hospital in Campus Martius, near Tiber, only for sick coachmen and carmen. Here, therefore, are entertained all the infirm of these professions; nor are they dismissed until they be known to have recovered their former health, so as to be able to drive their coaches or carts and govern their horses."

Passing to France, we find that country early distinguished by care for the sick poor. As in other countries in Europe, their monasteries formed the first hospitals. "Of hospitals which were not the direct offshoots of monasteries, the oldest, so far as we can tell, is still great and flourishing, the Hôtel Dieu (God's Hostelry), in Paris, founded by St. Landry, Bishop of Paris, at his own cost, in the year 600. This was in its original form a charitable organisation which embraced every form of aid to the poor and needy. Its domain was large and rich, and its administrators were men who stood high in the State." Some centuries later an extraordinary impulse was given to hospital work by the Crusades, not only by the importation of new diseases from the East, but by the religious zeal of the knights and pilgrims returning with new notions of medical science gained in other countries. This impulse of zeal showed itself in the formation of many religious orders vowed to the care of the sick. Of these, the most famous were the Knights of St. John of Jerusalem, commonly called the Knights Hospitallers, but in France other orders dedicated exclusively to works of healing did far better work than their more warlike brethren. The orders of St. Lazarus, St. Jacques du Pas, St. Vincent de Paul and others organised the care of the sick during the 12th and 13th centuries to a point nearly resembling the hospital systems of modern times. France then numbered 2,000 hospitals. But in the long civil wars the orders became weakened, the hospital endowments were appropriated, and the sick neglected. Isolated efforts were made by the French kings to reform the administration by removing dishonest officials and placing the direction of the Hôtel Dieu under the bourgeois, shopkeepers, and labourers in place of the clergy and nobility, but the abuses continued. At the beginning of the Revolution the number of hospitals in France had shrunk to 700, and the condition of many of them was appalling. It was not till this century that the thorough reforms were instituted which has placed France again foremost among the nations in works of healing.

In Germany hospital work dates principally from the Crusades. The Knights Templars and other warlike orders founded some of the earlier hospitals, but to the Order of the Holy Ghost, already alluded to as founded by Guido, of Montpellier, is due the systematic tending of the sick which, by the end of the 13th century, provided almost every town of importance in Germany with its "Hospital of the Holy Spirit." "The brethren of this order zealously studied medicine, and set high value upon medical science, and their foundations had a great influence in developing the new knowledge. While hospitals of earlier date were almost exclusively under spiritual control, and in times of confusion soon fell under the power of rapacious nobles or became almshouses, these, on the other hand, soon came to be managed in large measure by the towns, and to this circumstance is to be attributed the fact that they braved all the storms of time and flourish to this day in many parts of the Fatherland. Where they did not fall under municipal control they have perished." This unbroken continuity is a marked feature of the hospital work in many German towns. "Besides the numerous endowments, dating from the most ancient times which have in many places laid a financial foundation, there are societies which maintain their old connection with the care of the sick and infirm. The knightly Order of St. John is now merged in the evangelical nobility, and is still working for the same end. And the Catholic nobility has not lagged behind, and still in our own times does homage to these principles in the many habitations of the Knights of Malta." Almost all leprous hospitals in Germany were named after St. George, or St. Jürgen, and in Denmark, too, all the older hospitals bear the names of St. George's houses, showing their origin for leprous patients. It seems that in early days the Government seldom stirred in the care of the sick unless the sickness was infectious, and likely to be a source of danger to the State, but hospitals for plague, leprosy, small-pox, &c., were not infrequently founded by public authority, and the precaution of placing them outside the walls of the city was always observed.

In Spain the care of the sick early received attention, and three at least of their present hospitals show a history dating back to Moorish times. To San Juan de Dios and the order which he founded are due many of the Spanish hospitals, and the nursing in some of them is still carried on by this society. But in Spain, as in France, a period of decay suc-

* "Hospitals and Asylums of the World," 4 vols., and Portfolio of Plans. (Messrs. Churchill and the Scientific Press. 1893.)

ceeded to the impulses of zeal, and abuses, still in great part uncorrected, gradually crept into every branch of hospital administration.

Russia is principally noteworthy as being last in the field as regards hospital work. No definite hospital system existed in Russia until the establishment of the Boards of Charity by Catherine II. in 1775. The revenues of all charitable institutions were greatly increased by the Empress, whose generous example was followed by many wealthy persons.

In England our oldest hospitals are the work of private individuals. The earliest of which we have any record was founded by the Archbishop of Canterbury in 900. One of the oldest is St. Bartholomew's, founded between 1123 and 1133 by Rahere, the jester of Henry I.

We refer our readers for many most interesting details concerning this and other of our London hospitals to the volumes before us. It must suffice to say here that the general attitude of Londoners towards these institutions seems in two important particulars to have remained unchanged since their foundation, viz., boundless generosity on the occasion of any special need, and a sublime confidence in the administrators and indifference to the management of the funds during the years of repose.

